# Retained Acetylated Histone Four in Bull Sperm Associated With Fertility

**DOI:** 10.3389/fvets.2019.00223

**Published:** 2019-07-31

**Authors:** Muhammet Rasit Ugur, Naseer Ahmad Kutchy, Erika Bezerra de Menezes, Asma Ul-Husna, Bethany Peyton Haynes, Alper Uzun, Abdullah Kaya, Einko Topper, Arlindo Moura, Erdogan Memili

**Affiliations:** ^1^Department of Animal and Dairy Sciences, Mississippi State University, Starkville, MS, United States; ^2^Department of Genetics, School of Medicine, Yale University, New Haven, CT, United States; ^3^Department of Zoology, Pir Mehr Ali Shah-Arid Agriculture University, Rawalpindi, Pakistan; ^4^Warren Alpert Medical School of Brown University, Providence, RI, United States; ^5^Department of Pediatrics, Women and Infants Hospital of Rhode Island, Providence, RI, United States; ^6^Center for Computational Molecular Biology, Brown University, Providence, RI, United States; ^7^Department of Reproduction and Artificial Insemination, Selcuk University, Konya, Turkey; ^8^URUS Group LP, Madison, WI, United States; ^9^Department of Animal Science, Federal University of Ceará, Fortaleza, Brazil

**Keywords:** acetylation, epigenetics, histone 4, fertility, sperm

## Abstract

Bull fertility, ability of the sperm to fertilize and activate the egg and support embryo development, is vital for cattle reproduction and production. Even though majority of histones are replaced by protamines, some histones are retained in sperm. It is known that chromatin remodeling during spermatogenesis results in dynamic changes in sperm chromatin structure through post-translational modifications (PTM) of sperm histones, which are important for regulation of gene expression. However, amounts of sperm Histone 4 (H4), its acetylated form (H4 acetyl), and to what extent these molecular attributes influence sperm chromatin structure and bull fertility are unknown. These gaps in the knowledge base are important because they are preventing advances in the fundamental science of bovine male gamete and improvement of bull fertility. The objective of this study was to test the hypothesis that expression dynamics as well as PTM of sperm H4 are associated with bull fertility. Flow cytometry was utilized to quantify H4 and H4 acetylated form in sperm from seven high and seven low fertility Holstein bulls. The results indicated that the average number of cells with H4 or H4 acetyl expression in high and low fertility bull sperm were 34.6 ± 20.4, 1.88 ± 1.8, 15.2 ± 20.8, and 1.4 ± 1.2, respectively. However, the sperm enriched in both H4 and H4 acetyl were different between high and low fertility groups (3.5 ± 0.6; 1.8 ± 0.8; *P* = 0.043). The localization and detection of H4 and H4 acetylation were measured by immunocytochemistry which revealed that H4 and H4 acetylation were equally distributed in the sperm head of high and low fertility sires. Western blotting results confirmed the presence of the H4 and its acetylated form in the sperm. Bioinformatics studies demonstrated that H4 is highly conserved among mammalians, and have significant gene ontology on spermatogenesis, early embryo implantation, and sperm capacitation. The results are significant because it demonstrates the replacement of canonical histone H4 into modified H4 acetylation in sperm and regulate its dynamics which is crucial for bull fertility and reproductive biotechnology. These findings advance fundamental science of mammalian early development and reproductive biotechnology.

## Introduction

Bull fertility is an economically important trait and is defined as the ability of the sperm to fertilize and activate the egg, and to sustain embryo development which is crucial for efficient reproduction of cattle. Fertility is a complex trait with numerous determinants including molecular genetics, epigenetics, cellular and physiological aspects of sperm. Traditional semen evaluation techniques include analyses of sperm motility, membrane integrity and morphology to estimate bull fertility. However, they are both tedious and unreliable. As such, fertility differences among males cannot be accurately determined by these conventional methods (1). Identification of molecular, cellular and physiological biomarkers associated with fertilizing ability is vital for evaluation of semen quality and prediction of bull fertility. Sperm chromatin structure undergoes chromatin remodeling process including DNA methylation and post translational histone modifications to reach final maturation. Such modifications, comprise epigenetic profile of a sperm cell, and affect accessibility of male genome to maternal transcription factor in embryonic development (2, 3).

In mammals, chromatin of the sperm is highly compacted as compared to non-germinal cells. Sperm DNA is tightly coiled around the nucleohistone complex which includes histone H2A, histone H2B, histone 3 (H3), histone 4 (H4), and protamines (PRM) (3). During spermatogenesis, core histones are replaced by transition protein 1 (TP1) and TP2, and then transition proteins (TPS) are substituted by testis specific protamines (4, 5). Followed by sperm chromatin packaging into protamines, DNA is tightly coiled into a compact donut shape, also called as protamine toroid. However, nearly 15% percent of sperm still contain histones at final stage of the spermiogenesis in humans (6). Because of the histone retention in mice and humans (7), DNA bound to histones compared to protamines is less condensed, and paternal genes can interact with transcription factors easily. On the other hand, sperm DNA is more vulnerable to environmental stressors in de-condensation state because the condensed sperm head protects DNA from environmental damages. Additionally, hyperacetylation of H4 of core histones has a significant role in histone-to-protamine exchange (8).

Previous studies demonstrated that sperm chromatin damage reduces fertility in bulls (9), and abnormal protamination and H4 acetylation in human sperm are associated with subfertility (10). Additionally (7), reported that increased H3K27 trimethylation (H3K27me3) in sperm genome silenced gene promoters in early embryo. Moreover, H4 acetylation is related to success of the chromatin remodeling during spermatogenesis (11). Kutchy et al. (12) demonstrated the association between intensity of testis specific histone 2B and bull fertility. Furthermore, retained histones may epigenetically regulate gene expression in the early embryo (13). Lastly, our preleminary data suggested the possibility that H4 and acetylated H4 might be related to bull fertility (14). These data point to the importance of chromatin remodeling of histones and their PTM in fertility. However, amounts of H4, its posttranslational modifications, and to what extent these molecular attributes influence sperm chromatin structure and bull fertility are not well studied.

The objective of this study was to test the hypothesis that sperm H4 and its posttranslational modifications are associated with chromatin dynamics and bull fertility. Flow cytometry experiments were utilized to quantify H4 and acetylated H4 in bull sperm with different fertility scores. In addition, localization and presence of H4 and its acetylated form were determined using immunocytochemistry and Western blotting experiments, respectively. Moreover, computational biology and bioinformatic tools were applied to ascertain conservation of H4 across mammalian species and its interactomes and networks. The findings of the present study are significant because they help advance better understanding of how sperm histone H4 regulates fertility and mammalian development. The results also enhance fundamental science and technology of mammalian gamete and embryo development.

## Materials and Methods

### Experimental Design

Cryopreserved bull semen samples from mature Holstein bulls with reliable field fertility data were provided by Alta Genetics Inc. (Watertown, WI, USA), a leading animal breeding company. Semen straws from 14 Holstein bulls were used for flow cytometry experiment, and these samples were divided into two groups as high fertility (HF) and low fertility (LF) based on their fertility scores (Table 1). Bull sperm were used to determine the localization of H4 and H4 acetylation using immunocytochemsitry. Bull fertility scores were determined by Alta advantage Program (Alta Genetics, Inc., Watertown, WI, USA). The environmental and other determinants of fertility of sires were adjusted using threshold models which described in detail by Zwald et al. (15, 16). The average and standard deviation of conception rates of more than 300 individual Holstein bulls were calculated to determine fertility of each sire. Bulls that are performing at least two standard deviations above and below from average of entire population were classified as high fertile and low fertile, respectively. Selected HF and LF bulls account for outliers of population (17, 18).

**Table 1:** Fertility phenotypes of the Holstein bulls used for flow cytometry analysis.

**Bull number**	**Fertility status**	***In vivo* fertility score (% difference from average fertility)**	**Std of difference**	***P*-value**
1	HF	5.6	2.59	*P* > 0.05
2		5.0	2.3	
3		4.8	2.24	
4		4.6	2.12	
5		3.93	1.35	
6		3.79	1.3	
7		3.59	1.28	
8	LF	−3.7	−2.34	*P* > 0.05
9		−4.2	−2.6	
10		−5.49	−2.04	
11		−5.9	−2.73	
12		−6.3	−2.91	
13		−6.4	−2.94	
14		−7.3	−3.37	

*Bulls 1-7 were defined as high fertility (HF) and bulls 8-14 were grouped as low fertility (LF). Fertility of each bull was expressed as the percent difference of its conception rate from the average conception rate of all bulls. Probit.F90 software was used to estimate fertility. All bulls were individually represented with their in vivo fertility scores (p < 0.0001)*.

### Determining Levels of Sperm H4 and H4 Acetyl Using Flow Cytometry

Flow cytometry experiments were performed according to method described by Dogan et al. (19) and Kutchy et al. (12) with some modifications. Cryopreserved sperm samples from 14 Holstein bulls [7 high (HF) and 7 low fertility (LF)] thawed at 37°C for 30 s. Then, samples were centrifuged at 2,000 × g for 5 min to separate extenders. Remaining pellets were washed twice with PBS with 0.1% Bovine serum Albumin (BSA), then centrifuged at 2,000 × g at 4°C for 5 min. Following fixation in 1 ml of 4% formaldehyde, the samples were incubated at room temperature (RT) for 1 h in a separate centrifuge tube. Following centrifugation at 3,000 × g at 4°C for 5 min, pellets were resuspended in 250 μl of PBS and 250 μl of 0.1% triton X-100 in 0.1% sodium citrate in PBS as permeabilized solution on ice for 2 min, and then resuspended in washing buffer (WB: PBS with 0.1% Bovine Serum Albumin BSA) pellets were filtered. Final amount was divided into three aliquots and they were incubated with primary antibody at 4°C overnight. First group of samples were incubated with antibody against H4 (Mouse monoclonal Histone H4; Abcam, Cambridge, MA, USA; catalog # 31830; 1:200), and second group incubated with antibody against H4 acetylation (Rabbit monoclonal Histone H4 (acetyl K5 + K8 + K12 + K16); Abcam, Cambridge, MA, USA; catalog # 177790; 1:200). The third group was incubated with both antibodies against H4 and H4 acetylation. After overnight incubation, samples were centrifuged 3,000 × g at 4°C for 5 min, washed with 500 μl of washing buffer, and centrifuged 3,000 × g at 4°C for 5 min. Then, samples incubated with secondary antibodies for 2 h at RT as first incubation. Secondary antibodies were Donkey Anti-mouse FITC (Abcam, Cambridge, MA, USA; catalog # 97029; 1:250), and Donkey Anti-Rabbit DyLight 550 (Abcam, Cambridge, MA, USA; catalog # 96892; 1:250). After the incubation, samples were washed with washing buffer. Lastly, sperm samples were analyzed using BD- FACSCalibur flow cytometer (BD Bioscience San Jose, CA 95131–1807 USA). The experiments were repeated three times to find the percentage of sperm positive for H4, H4 acetylation, and double positive (H4 as well as H4 acetylation).

### Ascertain the Localization of H4 and H4 Acetyl Using Immunocytochemistry

Immunocytochemistry experiments were conducted according to protocols described by Lu et al. (20) and de Oliveira et al. (17) with some modifications. Cryopreserved sperm samples were thawed at 37°C for 30 s. Then, samples were washed with PBS including protease inhibitor (cOmplete; Roche, Indianapolis, IN, USA; catalog # 04693116001) and 10 mM ethylenediaminetetraacetic acid (EDTA). Next, the solution was incubated with 20 mM CHAPS for 20 min at RT and then, washed with PBS. Samples were incubated with 10 mM dithiothreitol (DTT) and 1 mg/ml of heparin solution at RT for 30 min in order to decondense sperm chromatin (21). Then sperm samples were fixed with 4% paraformaldehyde at 4°C for 10 min, and permeabilized with 2% Triton X-100 and 0.1% bovine serum albumin (BSA) in PBS at RT for 15 min. Following permeabilization, samples were washed using 50, 70, 95, and 100% ethanol. Later, fixation was finalized using 100% methanol for 20 min at −20°C. Samples were washed with a buffer solution (Washing Buffer (WB): PBS containing 0.1% Triton X-100) to remove methanol residues, and sperm cells were blocked with 1% BSA in the WB for 1 h at RT.

Samples were incubated with primary antibodies against H4 (Mouse monoclonal Histone H4; 1/100) and H4 acetylation (Rabbit monoclonal Histone H4 acetyl K5 + K8 + K12 + K16; 1/200) at 4°C overnight. Next day, samples were washed twice in WB at RT for 15 min. Then, samples were incubated with secondary antibodies against H4 (Donkey Anti-mouse, FITC) and H4 acetylation (Donkey Anti-Rabbit, DyLight 550) at RT for 1 h, and with 2.5 mg/ml of DAPI at RT for 10 min. The samples were examined under a confocal fluorescence microscope (Zeiss LSM 510) under 40X and 63X magnifications using immersion oil. Experiment was repeated to confirm the H4 and H4AC localization in bull sperm from high and low fertility bull three times.

#### Sperm Nuclear Protein Extraction for Western Blotting

Sperm H4 was extracted according to the methods of de Oliveira et al. (17) and Kutchy et al. (12). Briefly, cryopreserved semen samples from three high fertility and three low fertility bulls thawed at 37°C for 30 s, and samples were washed twice in PBS with protease inhibitor. Samples including 5 ×10^6^ cells were washed twice with 400 ml of 1 mM phenylmethylsulphonyl fluoride (PMSF) in ddH_2_O to lyse the cells. Next, 100 ml of 20 mM EDTA, 1 mM PMSF, and 100 mM Tris (pH 8.0) were added to the pellets followed by the addition of 100 ml of 6 M guanidine hydrochloride, 575 mM DTT, and 200 ml of 552 mM sodium iodoacetate. Then, samples were incubated at 20°C for 30 min by protecting from light. Additionally, 1 ml of cold ethanol (−20°C) was added to each sample, and these samples were incubated at −20°C for 3 min and centrifuged at 12,000 × g at 4°C for 10 min. Following one more wash with ethanol, pellet was resuspended in 1 mL of 0.5 M HCl and incubated at 37°C for 15 min and centrifuged at 10,000 × g at 25°C for 10 min. After centrifugation, supernatant was kept, and 300 μL of 100% trichloroacetic acid (TCA) to a final concentration of 20% TCA was added to precipitate nuclear proteins. Following 5 min incubation at 4°C, the samples were centrifuged at 12,000 × g for 10 min. The protein pellet was washed twice with 500 ml of 1% 2-mercaptoethanol in acetone, dried, and stored at −30°C.

#### Western Blotting Analysis

Protein concentration in samples was determined using Quick Start™ Bradford Protein Assay Kit 2 (Bio Rad®, Hercules, CA, USA; catalog # 5000202). Sperm nuclear proteins were precipitated using cold acetone at −20°C for 3 h. After centrifugation, supernatant was removed, and pellet was resuspended in 50 μl of 1x Laemmli sample buffer (Bio Rad®, Hercules, CA, USA) with 5% 2- mercaptoethanol, and boiled at 95°C for 10 min. Ten microgram of aliquots from this sample were taken, and sperm nuclear proteins were separated using a vertical polyacrylamide gel electrophoresis (4–20% SDS-PAGE; Mini-Protean TGX™ gel). Next, protein bands were transferred from gels to Immobilon®-P polyvinylidene difluoride (PVDF) membrane using HEP-1 semi-dry electro blotting (Thermo-Scientific Inc.® Waltham, MA USA) set at 46 mA for 2.5 h. To block binding sites, membrane was incubated in 5% BSA in PBS-0.1% Tween 20 (PBS-T) at RT overnight. Next day, membrane was washed with 0.1% Tween 20 in PBS for 10 min.

For Histone 4, samples were incubated with primary antibody against H4 (Mouse monoclonal Histone H4, Abcam®, Cambridge, MA, USA; catalog #31830; 1/1,000 dilution) with 0.1% Tween 20 in PBS for 3 h at 37°C. Then membranes were washed three times for 10 min with 0.1% Tween 20 in PBS. The membranes were incubated with secondary antibody (Donkey Anti-mouse FITC; 1:1,000), Strep Tactin -HRP conjugate (Bio Rad®, Hercules, CA, USA, catalog #1610380; 1:10,000), and internal loading control (c-Myc Antibody, Santa Cruz, Dallas, Texas, USA; catalog #4084, 200 μg/ml) for 90 min at 37°C. For H4 acetylation, primary antibody was Anti- Histone H4 (acetyl K5 + K8 + K12 + K16) (Abcam®, 2: 20,000), and secondary antibody was Donkey Anti-Rabbit DyLight (Abcam®, 1: 2,000). After incubation with secondary antibody, membranes were washed three times with 0.1% Tween 20 in PBS for 10 min. The bands were revealed using a chemiluminescence reagent (Clarity™ Western ECL Substrate, Bio-rad®, Hercules, CA, USA) and Image Laboratory software (Bio-Rad®) for 30 s.

### Bioinformatics

Clustal Omega software (https://www.ebi.ac.uk) (22) was used to ascertain multiple sequence alignment of H4. The domain structure of conserved areas of H4 was investigated using NCBI's conserved domain database (http://www.ncbi.nlm.nih.gov/Structure/cdd/cdd.shtml) (23). Functional motifs of H4 were determined using GenomeNet bioinformatic tool (https://www.genome.jp) (24) and UniPort software (https://www.uniprot.org) (25). The STRING®software (https://string-db.org/) was used to investigate biological networks of sperm H4 (26).

### Statistical Analysis

The intensity of H4 and H4 ACETYL were assessed using mixed model analysis using PROC MIXED with SAS for Windows 9.4 (SAS Institute, Inc., Cary, NC). Group was included as a fixed effect with bull as a random effect. Accordingly, separate models with fertility score as the fixed effect were fit for both the high and low fertility groups to better understand the relationship between fertility score and H4 intensity, H4 ACETYL intensity, and H4 and H4 ACETYL, respectively. Box plots and regression line plots were made using PROC SGPLOT for H4 intensity, H4 ACETYL intensity, and double positive (H4 and H4 ACETYL) intensity. The regression line was determined using the model-predicted intensity values for each of fertility score using the mixed effects model. A scatter plot of the raw, unadjusted data points was superimposed on the regression line plot. Conditional residual plots were used to assess model fit. An alpha level of 0.05 was used to determine statistical significance.

## Results

### Expression Dynamics of H4 and H4 Acetyl in Bull Sperm Using Flow Cytometry

**Figure 1:**
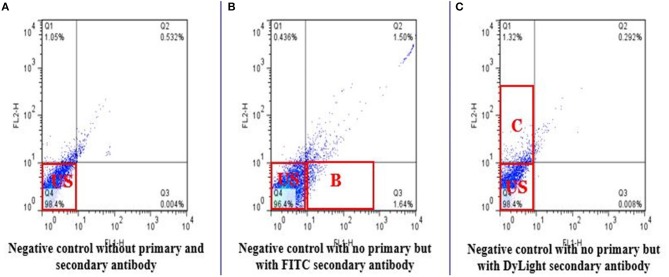
Flow cytometric measurements of negative controls. The negative controls demonstrate absence of fluorescence. FL1-H detects FITC and FL2-H detects DyLight 550 in stained sperm. **(A)** Do not include any secondary antibody. Q4 represents unstained sperm. **(B)** Only FITC used was as a secondary antibody to target Histone 4 (Q3). **(C)** Only DyLight was used as a secondary antibody to target Histone 4 acetylation on lysine 5, 8, 12, 16 (Q1).

**Figure 2:**
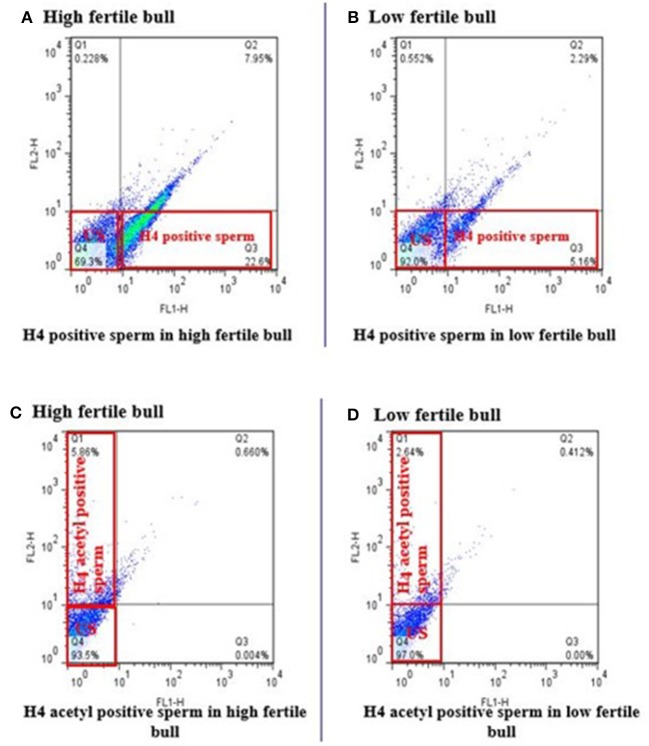
Flow cytometric measurement of H4 and H4 acetyl in high and low fertility bulls. The FL1-H channel (Q3 quadrant) shows the florescence of H4 positive sperm and US (Q4 quadrant) unstained sperm or not positive for H4 in high and low fertile bulls **(A,B)**. Similarly, FL2-H channel (Q1 quadrant) are H4 acetyl positive sperm and US (Q4 quadrant) unstained sperm or not positive for H4 acetyl in high and low fertile bull **(C,D)**.

**Figure 3:**
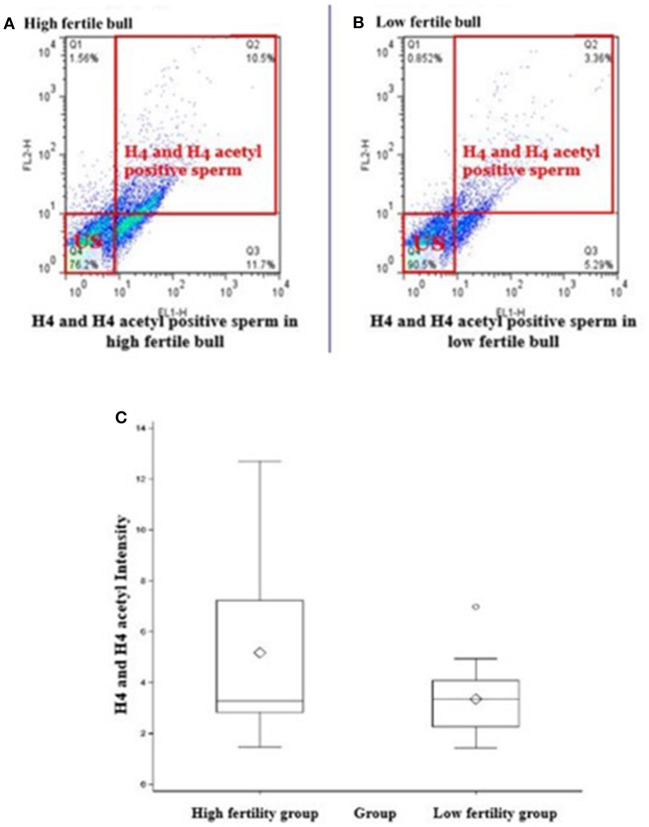
Flow cytometric measurements of sperm H4 and H4 acetyl. Differences of the H4 levels in sperm from HF and LF groups are uncovered. **(A,B)** Represent high or low fertility bulls with H4 and H4 acetyl positive sperm. **(C)** Boxplot depicts the difference between H4 and H4 acetyl positive sperm in high or low fertility bulls (3.5 ± 0.6; 1.8 ± 0.8; *P* = 0.043).

Total of 100,000 sperm per bull per repeat were analyzed to quantify H4 and H4 acetyl in sperm from seven high and seven low fertility Holstein bulls (Table 1). The flow cytometer was setup by analyzing controls with no secondary antibody, with FITC (captured by FL1-H channel) secondary antibody only and DyLight 550 (captured by FL2-H channel) secondary antibody (Figures 1A–C). The results indicated that percentage of sperm tagged by H4 (FL1-H channel) among high and low fertility bulls when averaged were 34.6 ± 20.4, 15.2 ± 20.8, respectively (Figures 2A,B). Similarly, the percentage of sperm tagged by H4 acetyl (FL2-H channel) among high and low fertility bulls when averaged were 1.88 ±1.8 and 1.4 ± 1.2, respectively (Figures 2C,D). These values, however, were not different between high and low fertility group (*p* > 0.05). On the other hand, the percentage of sperm tagged by both H4 and H4 acetyl (double positive) among high and low fertility bulls when averaged were (3.5 ± 0.6; 1.8 ± 0.8) (Figures 3A–C) and were different (*p* = 0.043).

### Ascertain the Localization of H4 and H4 Acetyl by Using Immunocytochemistry

**Figure 4:**
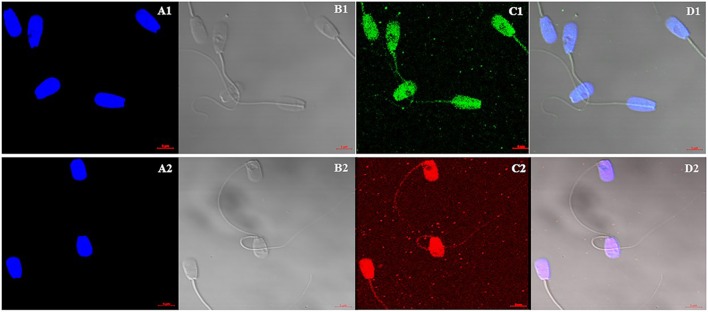
Cellular localization of Histone 4 and Acetylated H4 in bull sperm using confocal microscopy. **(A1,A2)**: DNA stained with DAPI; **(B1,B2)**: Bright field image; **(C1)**: H4 stained with FITC; **(C2)**: Acetylated H4 stained with DyLight; **(D1,D2)**: Merged Images of DAPI, bright field and FITC. H4 and H4 acetyl well distributed in the head area of sperm. There were no differences between high and low fertility bulls in terms of distribution and signal intensity.

Immunocytochemistry method was performed to find the localization and patterns of H4 (Figures 4A1–D1) and H4 acetyl (Figures 4A2–D2) in sperm from higher and lower fertility bulls. Both H4 and H4 acetyl well distributed in the head area of sperm (Figures 4A,D).

### Expression of H4 and H4 Acetyl in Bull Sperm Using Western Blotting

**Figure 5:**
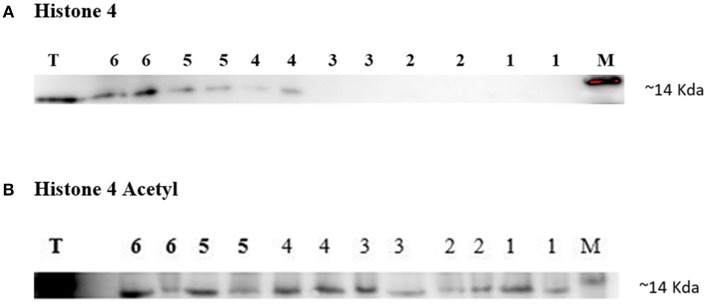
Immunoblotting of Histone 4 and Acetylated H4 from high and low fertility bulls. Results from the HF bulls are represented in lanes 1–3 and from the LF bulls in lanes 4–6 **(A,B)**. Each sample was loaded in duplicate for technical repeat purpose **(A,B)**. Bull testis (T) was used as positive controls. c-Myc was used as a loading marker (M).

Western blotting technique was used to detect the expression of H4 and H4 acetyl in the sperm from high and low fertility bulls. Results revealed that the H4 and acetylated H4 were detectable in sperm from all of bulls (Figures 5A,B).

### Bioinformatic Analyses of H4

**Figure 6:**
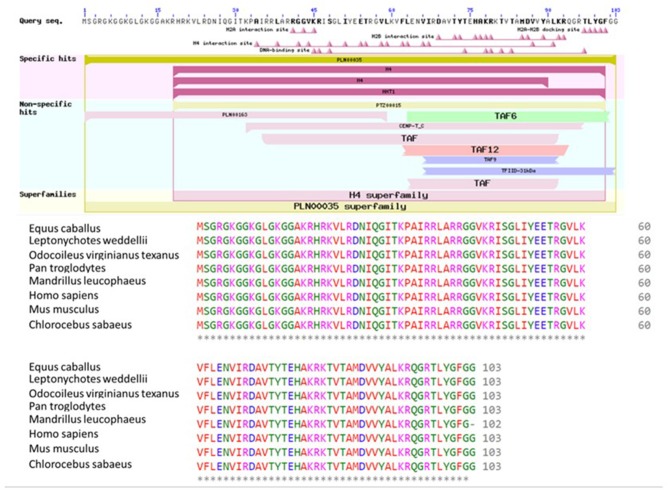
Conserved domains on H4 [*Bos taurus*], and multiple sequence alignment of H4 using *Bos taurus* as a query sequence. Conserved domain structure on H4 [*Bos taurus*] (ref: XP_024839710.1) using NCBI's domain search tool. Conserved sequences of H4 among eight species were determined using Clustal Omega software. Starred regions show 100% matches.

**Figure 7:**
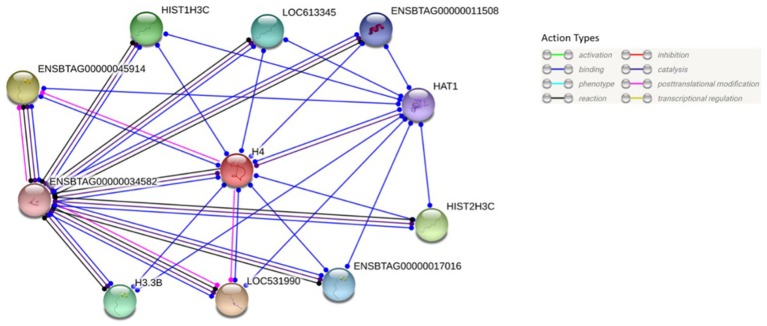
Protein-protein interaction analysis of H4. Protein-protein interactions of H4 were analyzed using web-based STRING®software. Its predicted functional partners are LOC531990 (Histone H3), ENSBTAG00000045914 (Histone H3), HIST2H3C (Histone cluster 2), HIST1H3C (Histone H3.1), H3.3B (Histone H3.3), LOC613345 (Uncharacterized protein), ENSBTAG0000001701 (H3 histone, family 3C), ENSBTAG00000011508 (Histone H3.3C), HAT1 (Histone acetyltransferase type B catalytic subunit), ENSBTAG00000034582 (Histone H2B type 2-E).

BLASTP online software (27), provided by NCBI, was used to search H4 proteins from *Bos taurus* sequence. Protein sequences of 20 mammalian were used for multiple sequence alignment. Clustal Omega research showed that H4 sequence were 100% conserved among *Equus caballus, Leptonychotes weddellii, Odocoileus virginianus texanus, Pan troglodytes, Homo sapiens, Mus musculus, Chlorocebus sabaeus*, and *Bos taurus* (Figure 6) GenomeNet and UniPort bioinformatics tools were used to determine functional motifs of H4. Bovine H4 have significant gene ontology on a number of biological events, such as sperm capacitation, methylated histone binding, and histone acetyl transferase regulator activity (Table 2). The biological networks of H4 are shown in Figure 7. Additionally, ORC3 (Origin recognition complex subunit 3) and ORC4 (Origin recognition complex subunit 4) binds histone H3 and H4 trimethylation marks H3K9me3, H3K27me3, and H4K20me3. Moreover, ING4 (Inhibitor of growth protein 4) is responsible for H4 acetylation *in vivo*.

**Table 2:** A summary of the enriched gene ontology terms of Histone 4.

**Gene ontology term name**	**Gene ontology ID**
Establishment of Sertoli cell barrier, spermatogenesis	[GO:0007283]
Embryo implantation	[GO:0007566]
Negative regulation of chromosome condensation	[GO:1902340]
Oogenesis	[GO:0048477]
Single fertilization	[GO:0007338]
Spermatid development	[GO:0007286]
Embryonic digit morphogenesis	[GO:0042733]
In utero embryonic development	[GO:0001701]
Positive regulation of histone H3-K4 methylation	[GO:0051571]
Positive regulation of luteinizing hormone secretion	[GO:0033686]
Seminiferous tubule development	[GO:0072520]
Pri-miRNA transcription by RNA polymerase II	[GO:0061614]
Positive regulation of histone H3-K9 acetylation	[GO:2000617]
Histone H4-K20 trimethylation	[GO:0034773]
Histone H2A monoubiquitination	[GO:0035518]
Establishment of Sertoli cell barrier	[GO:0097368]
Sperm capacitation	[GO:0048240]
Methylated histone binding	[GO:0035064]
Histone H3-K4 trimethylation	[GO:0080182]
Spermatid nucleus differentiation	[GO:0007289]
Male germ-line stem cell asymmetric division	[GO:0048133]
Histone acetyltransferase regulator activity	[GO:0035034]
Post-embryonic development	[GO:0009791]
Embryonic placenta development	[GO:0001892]
DNA repair	[GO:0006281]
Histone lysine demethylation	[GO:0070076]
Spermatid nucleus differentiation	[GO:0007289]
Oocyte maturation	[GO:0001556]
Uterus morphogenesis	[GO:0061038]
Histone methyltransferase activity (H3-K36 specific)	[GO:0046975]
Sperm motility	[GO:0097722]
Negative regulation of histone H3-K14 acetylation	[GO:0071441]
Embryonic process involved in female pregnancy,	[GO:0060136]
Negative regulation of histone H3-K9 trimethylation	[GO:1900113]
Negative regulation of histone phosphorylation	[GO:0033128]
Negative regulation of helicase activity	[GO:0051097]

*GenomeNet and UniPort bioinformatics tools were used to determine functional motifs of H4*.

## Discussion

Histone-to-protamine exchange is one of the most crucial steps in sperm chromatin remodeling because this process determines the degree of chromatin condensation which is essential for fertilization. It is thought that hyperacetylation of core histones is important for correct histone-protamine exchange (10, 20). The purpose of the present study was to test the hypothesis that expression dynamics of H4 as well as its PTM are associated with bull fertility. Flow cytometry, immunocytochemistry, Western blotting, and bioinformatic approaches were employed to test such hypothesis.

We demonstrated that expression of sperm H4 and acetylated H4 were more abundant in high fertility bulls as compared to those of low fertility bulls, with 3.5 ± 0.6 and 1.8 ± 0.8 respectively. However, the average numbers of sperm cells counted with H4 or Acetylated H4 were not significantly different among high and low fertility bulls. Immunocytochemistry results demonstrated that sperm H4 and acetylated H4 localized in sperm head, and it distributed homogenously. Also, there were no differences between high and low fertility bulls in terms of signal intensities and localizations of H4 and acetylated H4. Bioinformatics analyses revealed that amino acid sequences of H4 were completely conserved across the 20-mammalian species indicating the evolutionary conservation of the propagation of species at the molecular and cellular levels. In addition, H4 has a significant gene ontology on spermatid development, oogenesis, sperm motility, embryonic placenta development, male germ-line stem cell asymmetric division, sperm capacitation, spermatid differentiation, chromatin remodeling, and nucleus organization.

Previous studies demonstrated that histones and their posttranslational modifications play a significant role in sperm DNA packaging (3), molecular morphology and functions (17). Hyperacetylation of H4 is a critical step on histone removal process (10). Acetylation changes overall charge of histone tail from positive to neutral, thus the interaction between negatively charged phosphate group of DNA and N termini of histones is reduced (28). Following the acetylation process, core histones are replaced by transition nuclear proteins and protamines. Such chromatin reorganization steps make sperm chromatin more compact which is important for sperm motility and fertilization (6).

However, retained H4 makes sperm cells less compact and more susceptible disturbing factors through the movement of sperm from testis to female reproductive tract. Aoki et al. (29) reported that abnormal protamine: histone ratio cause sterility in human. Even though, there is some general information of sperm H4 and their attributions on fertility and sperm structure, specific contributions on reproduction is not well-known. The results reported in this study clarified contributions of sperm H4 and acetylated H4 on reproduction. As such, we propose that reduced levels of hyperacetylated H4 in sperm from low fertility bulls are associated with loose of chromatin structure leading to abnormalities in sperm molecular morphology and function.

In conclusion, the results of this study are significant because they illuminate epigenetic changes in bull sperm that enhances both the fundamental science and technology of bull fertility and cattle reproduction. The molecular and cellular markers can be used to evaluate semen quality and predict bull fertility. Because of the similarities both in genetics and physiology between bulls and other mammalian males, the data and knowledge produced in this study are expected to contribute to the advancement of basic science and biotechnology of other mammals including humans and endangered species.

## Data Availability

All of the data generated and analyzed in this study are included in the manuscript as tables and figures. The corresponding author will respond to the queries regarding the raw data and reasonable accommodations will be provided.

## Author Contributions

This study was conceptualized by NK, AK, AM, and EM. Data were curated by MU, NK, and EBM. Investigations were carried out by MU, NK, EBM, AU-H, AU, and BH under supervision of EM. Essential samples and phenotypic data were provided by AK and ET. Original draft was written by MU, NK, EBM, BH, and EM. Reviews and editing were completed by NK, MU, and EM.

### Conflict of Interest Statement

ET was employed by company URUS Group LP. The remaining authors declare that the research was conducted in the absence of any commercial or financial relationships that could be construed as a potential conflict of interest.
